# DBSegment: Fast and robust segmentation of deep brain structures considering domain generalization

**DOI:** 10.1002/hbm.26097

**Published:** 2022-10-17

**Authors:** Mehri Baniasadi, Mikkel V. Petersen, Jorge Gonçalves, Andreas Horn, Vanja Vlasov, Frank Hertel, Andreas Husch

**Affiliations:** ^1^ National Department of Neurosurgery, Centre Hospitalier de Luxembourg Center for Systems Biomedicine, University of Luxembourg Esch‐sur‐Alzette Luxembourg; ^2^ Department of Clinical Medicine, Center of Functionally Integrative Neuroscience University of Aarhus Aarhus Denmark; ^3^ Luxembourg Center for Systems Biomedicine University of Luxembourg Esch‐sur‐Alzette Luxembourg; ^4^ Neuromodulation and Movement Disorders Unit, Department of Neurology Charité–Universitätsmedizin Berlin Berlin Germany; ^5^ MGH Neurosurgery and Center for Neurotechnology and Neurorecovery at MGH Neurology Massachusetts General Hospital Harvard Medical School Boston USA; ^6^ Center for Brain Circuit Therapeutics, Department of Neurology, Brigham and Women's Hospital Harvard Medical School Boston USA; ^7^ National Department of Neurosurgery Centre Hospitalier de Luxembourg Luxembourg

**Keywords:** confounder, deep brain structures, deep learning, magnetic resonance imaging, segmentation

## Abstract

Segmenting deep brain structures from magnetic resonance images is important for patient diagnosis, surgical planning, and research. Most current state‐of‐the‐art solutions follow a segmentation‐by‐registration approach, where subject magnetic resonance imaging (MRIs) are mapped to a template with well‐defined segmentations. However, registration‐based pipelines are time‐consuming, thus, limiting their clinical use. This paper uses deep learning to provide a one‐step, robust, and efficient deep brain segmentation solution directly in the native space. The method consists of a preprocessing step to conform all MRI images to the same orientation, followed by a convolutional neural network using the nnU‐Net framework. We use a total of 14 datasets from both research and clinical collections. Of these, seven were used for training and validation and seven were retained for testing. We trained the network to segment 30 deep brain structures, as well as a brain mask, using labels generated from a registration‐based approach. We evaluated the generalizability of the network by performing a leave‐one‐dataset‐out cross‐validation, and independent testing on unseen datasets. Furthermore, we assessed cross‐domain transportability by evaluating the results separately on different domains. We achieved an average dice score similarity of 0.89 ± 0.04 on the test datasets when compared to the registration‐based gold standard. On our test system, the computation time decreased from 43 min for a reference registration‐based pipeline to 1.3 min. Our proposed method is fast, robust, and generalizes with high reliability. It can be extended to the segmentation of other brain structures. It is publicly available on GitHub, and as a pip package for convenient usage.

## Graphical abstract

We provide a fast, accurate, and easy‐to‐use method for the segmentation of deep brain structures, with a focus on small structures not covered in previous studies. We validate our method on a large diverse dataset, including clinical data with high variability, explicitly considering potential biases induced by the training data. We interpret our results through ablation studies and provide the trained network as a pip package.

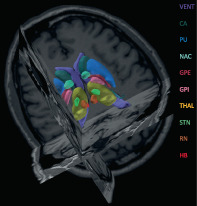

## 1 INTRODUCTION

Segmentation of deep brain nuclei from structural Magnetic Resonance Imaging (MRI) data is widely used in clinical practice and in research (Heckemann et al., 2010; Milletari et al., 2017). Segmentation allows localization of structures and extraction of morphological features, relevant for diagnosis, treatment planning, and disease follow‐up (Helms et al., 2009; Pham et al., 2000). For example, in Parkinson's Disease (PD), where patients show characteristic morphological changes of the Substantia nigra (SN), segmentation of this region and other basal ganglia nuclei has been important in studying disease progression. Furthermore, accurate segmentation processes are of value for identifying diagnostic biomarkers that can help differentiate PD from other parkinsonian syndromes (Bae et al., 2021; Basukala et al., 2021).

Another important application is preoperative planning and postoperative evaluation of deep brain stimulation (DBS) surgery (Horn & Kühn, 2015; Middlebrooks et al., 2018; Reinacher et al., 2019). DBS is an established treatment for movement disorders and psychiatric diseases, in which the target, a specific deep brain structure, is stimulated via an implanted electrode (Abelson et al., 2005; Benabid, 2003; Herzog et al., 2003; Larson, 2014). DBS target structures cannot be clearly visualized during the intervention and therefore, preoperative localization of the target structure is important for the surgical targeting (Dergachyova et al., 2018). Precise planning is necessary for accurate placement of the DBS electrodes, and results in a better treatment outcome (Wang et al., 2016). After surgery, the device settings, such as stimulation amplitude, and the active contacts of the implanted electrode, are systematically tested during a programming session (Pavese et al., 2020). The goal of this session is to find optimal settings that stimulate the target region, while avoiding regions that cause side‐effects. A detailed understanding of the spatial relationship between target structure, region of avoidance, and the implanted electrode, can provide valuable information for the time‐consuming process of fine‐tuning stimulation parameters (Anderson et al., 2018, Cubo et al., 2019, Åström et al., 2018).

### 1.1 Segmentation by registration

For brain structure segmentation, the most used method is registration‐based, also called atlas‐based registration (Van Der Lijn et al., 2012), using an atlas containing labelled segmentations in a specific template imaging‐space. The reference template data is typically an averaged T1‐weighted (T1w) image such as Montreal Neurological Institute *(MNI)*, *Deepbrain7T*, or the California Institute of Technology (CIT168) template (González‐villà et al., 2016, Fonov et al., 2009, Lau et al., 2017, Pauli et al., 2018). This method is typically multistage, including preregistration steps, such as bias field correction and skull stripping, followed by multiple registration stages, with increasing degrees of freedom as the registration advances (Vogel et al., 2020; Wang et al., 2014). The subject's image is registered to the template, and the atlas (e.g., Distal, THOMAS, and the CIT168 atlas, etc.) is used to map the location of the labelled brain structures (Ewert et al., 2018; Pauli et al., 2018; Su et al., 2019). The final output is structural segmentation maps generated in the subject‐specific‐image‐space. Multispectral approaches that include other sequences beyond T1 have been shown to lead to better results (Ewert et al., 2018). Popular registration tools include Advanced Normalization Tool (ANTs^1^), FMRIB Software Library (FSL^2^), Statistical parametric mapping (SPM^3^), and Deformable registration via attribute matching and mutual‐saliency weighting (DRAMMS) (Andersson et al., 2010; Ashburner, 2007; Ashburner & Friston, 2005; Ashburner & Friston, 2011; Avants et al., 2009; Jenkinson et al., 2002; Jenkinson & Smith, 2001; Ou et al., 2011).

The accuracy of the segmentation can be impacted by preprocessing steps, registration method and algorithm parameter selection (Vogel et al., 2020). A number of studies have addressed the challenge of segmentation accuracy, and have proposed registration‐based pipelines for brain structure segmentation (Feng et al., 2017; Wang et al., 2014). For example, Schönecker et al proposed an automated pipeline for linear registration of subject images to MNI space using FSL tools. Skull‐stripped images, registered to MNI space with an affine transformation, are refined further in a multistage process using first a subcortical mask extended to cover potential enlarged ventricles, and second a smaller stereotactic mask, covering the basal ganglia (Schönecker et al., 2009). DBS Auto Report (DBSAR) is another automatic pipeline that maps basal ganglia structures from the Deep7T atlas to the subject's image. It is based on a multistage ANTs registration pipeline (Husch et al., 2018). Another example of a tool integrating multiple registration approaches is Lead‐DBS, a DBS‐focused toolbox that provides a user‐interface to perform each registration step with user‐selected method. This toolbox supports a large number of well‐established registration algorithms (Horn et al., 2019; Johnson et al., 2007).

In multistage registration pipelines, errors can occur at different stages and lead to incorrect or inaccurate segmentation maps. During the registration process, the subject's image is mapped to the reference template image. Cases where there are significant differences between subject and template images can especially challenge the registration process. In clinical settings, this can present a significant challenge, for example if a patient's image has a reduced field of view (a partial brain), or in the case of head tilting during the image acquisition (Greve & Fischl, 2009). Furthermore, the registration process includes optimization steps and is computationally demanding, especially in the case of a multi‐atlas registration. Therefore, there is a clear need for computationally less demanding methods, for clinical use, or indeed for large cohort studies. (Cabezas et al., 2011, González‐villà et al., 2016, Henschel et al., 2020).

### 1.2 Deep learning based segmentation

Supervised deep learning approaches are an alternative for brain structures segmentation (Akkus et al., 2017). Several existing studies have proposed segmentation of brain structures using convolutional neural networks (CNN) (Bao et al., 2016; Brebisson et al, 2015; Kushibar et al., 2018). For example, Quick Segmentation of NeuroAnaTomy (QuickNAT) segments the whole brain into 27 structures. It consists of three 2D fully CNNs (F‐CNNs) for the axial, coronal, and sagittal planes. During the inference, the three orthographic planes are aggregated. The F‐CNNs have U‐Net architecture with unpooling layers, and dense connections (Roy et al., 2019). FastSurfer segments the brain into 95 regions. Similar to QuickNAT, it consists of three 2D F‐CNNs for the three orthographic planes followed by a view aggregation step. The F‐CNNs have U‐Net architecture with competitive dense blocks, multi‐slice information, and competitive skip pathways (Henschel et al., 2020). SynthSeg proposes a CNN trained on synthetic data (Billot et al., 2021). The intensity of the synthetic images are sampled from a uniform distribution instead of normal distributing. Therefore, the training images have fully random intensity values, and the network trained on these data are agnostic to contrast and resolution. However, these methods focus on the segmentation of large brain structures and do not include smaller deep brain structures.

Hough‐CNN, proposed a method for segmenting 26 deep brain structures, where the second last fully connected layer of the CNN is used to localize and segment the structures (Milletari et al., 2017). The method was tested and evaluated on 26 subjects (122 volumes). However, performance was poor when compared to the methods using U‐Net architecture, the current state‐of‐the‐art for segmentation. A publication called M‐net, proposed a network for segmenting 14 structures using a 2D‐U‐Net architecture (Mehta & Sivaswamy, 2017). This method was tested on two datasets, International Brain Segmentation Repository (IBSR) with 18 subjects, and MICCAI 2013 SATA challenge, with 35 subjects for training and 12 for testing. Rashed et al. proposed a method for segmenting 7 deep brain structures using a single‐encoder, and multi‐decoder CNN (Rashed et al., 2020). The method was evaluated on two datasets, NAMIC (Brain Multimodality) dataset with 18 subjects, and the MICCAI 2012 workshop dataset with 35 subjects. However, for all of these methods (Mehta & Sivaswamy, 2017; Milletari et al., 2017; Rashed et al., 2020), no testing on unseen datasets or validations were performed, which is an important step to ensure generalizability to unseen data. To us, this was of particular importance, considering the high degree of variability in MRI data from different datasets.

### 1.3 Our proposed method

**FIGURE 1:**
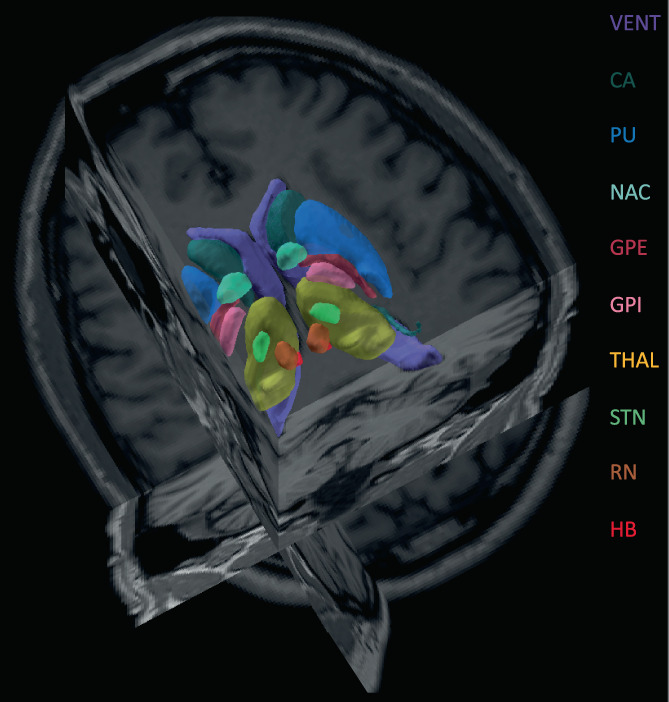
Deep brain structures segmentation. The segmentation results of the network are shown for one of the test scans. The right and the left hemisphere are visualized with the same color. SNC, SNR, VIM, and VPL are not shown in this figure. The full name of the structures can be found in Table 2

In this study, We propose deep brain structures segmentation (DBSegment), a rapid deep learning approach to segment 30 deep brain structures from T1w MRI (Figure 1).The method consists of a preprocessing step to transform all the images to the same reference orientation followed by nnU‐Net framework for segmentation (Isensee et al., 2021), and finally, transformation back to the native space, all in one step. The preprocessing helps the network to generalize even on the datasets where nnU‐Net fails (Isensee et al., 2021).

**FIGURE 2:**
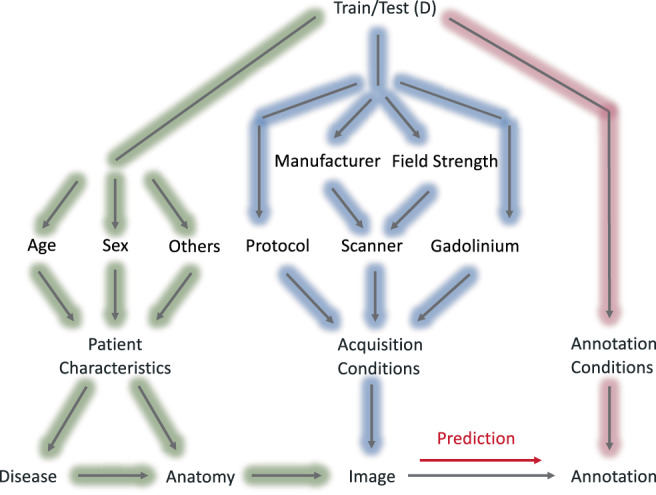
The causal diagram of the deep brain structures segmentation workflow. The factors affecting the annotation prediction are categorized to three main groups; patient characteristics (green), acquisition conditions (blue), and annotation conditions (pink)

We test our fully automated method, using data from both research‐oriented and clinical acquisitions, with imaging data from different scanners, sequence protocols, age range, subject health, and sex. We compared our approach to a multistage registration‐based method. We evaluate the generalizability of the network by performing a leave‐one‐dataset‐out (LODO) cross‐validation, and extensive testing on an unseen multicenter test set. Finally, we perform an ablation study to evaluate the cross‐domain transportability, by studying different factors affecting the robustness of the deep leaning models on clinical data. In this work, we focus on the small deep brain structures, such as ventral intermediate thalamic nucleus (VIM), ventral posterolateral nucleus of thalamus (VPL), and the habenular nuclei (HN), which have not been the focus of previous methods development.

## 2 METHODS

We segment 30 deep brain structures from T1w MRIs with a deep learning‐based method using the nnU‐Net platform (Isensee et al., 2021). We used a large and diverse dataset, annotated using a registration‐based method. Our method involves an essential preprocessing to conform all MR images to the same orientation, voxel spacing, and dimension, before the network training. We trained and evaluated the network with the LODO cross‐validation, and performed testing on several unseen datasets. Additionally, we performed an ablation study by separating results into different domains based on the factors affecting the segmentation performance. This was done to evaluate the cross‐domain transportability. Finally, we performed another ablation study to emphasize the importance of the preprocessing steps.

### 2.1 Datasets

We used data from 10 public datasets together with 4 anonymized datasets from clinical centers for training and testing. The following datasets were used for training and LODO cross‐validation, the Human connectome project's young adults, HCP^4^ (Van Essen et al., 2012), Autism Brain data exchange II, ABIDE II^5^ (Di Martino et al., 2017), Parkinson's Progression Markers Initiative, PPMI^6^ (Aleksovski et al., 2018), Alzheimer's disease Neuroimaging Inititive, ADNI^7^ (Mueller et al., 2005), Open Access Series of Imaging Studies 3, OASIS3^8^ (LaMontagne et al., 2019), Minimal Interval resonance Imaging in Alzheimer's Disease, MIRIAD^9^ (Malone et al., 2013), and deidentified MRIs of Epilepsy patients from Shanghai Ruijin Hospital, SRH (clinical). The method is validated using data from unseen datasets (test sets), including, Information eXchange from Images, IXI,^10^ the UCLA Consortium for Neuropsychiatric Phenomics, LA5c,^11^ obtained from the OpenfMRI database, with accession number of ds000030 (Poldrack et al., 2016), Designed Database of MR Brain Images of Healthy Volunteers from the University of North Carolina, UNC^12^ (Bullitt et al., 2005), Designed Database of MR brain images of Healthy Volunteers, Travelling Human phantom, THP^13^ (Magnotta et al., 2012), and deidentified data of the DBS patients from the Centre Hospitalier de Luxembourg, CHL (clinical), Charité Universitätsmedizin Berlin, CUB (clinical), and Aarhus University Hospital in Denmark, AUH (clinical). We collected a diverse dataset, including images from various scanners, with different acquisition protocols, and intensity ranges. The data contains healthy subjects and patients with neurological and psychiatric disorders across a wide range of age. The Maximum number of subjects used from one dataset is 100 (randomly selected), to avoid overfitting to one specific dataset. All datasets except THP, contain only a single scan from each subject. Detailed information about the training, and test data can be found in Table 1.

**TABLE 1:** The meta data of the training and the test set. Seven datasets are used for the training and the leave one dataset out cross‐validation, and the other seven datasets are used for testing

Usage	Dataset	Scanner	1.5 T/3 T	Protocol	Gado	Disease	Origin	Age	Counts
Training	HCP	SM	3 T	MPRAGE	N	HT	RS	22–35	100
A	OASIS3	SM	3 T	MPRAGE	N	HT, AD, DM	RS	49–87	100
Cross	ADNI	GE, SM, PL	1.5 T, 3 T	MPRAGE	N	HT, AD, MC, CI	RS	57–90	100
Validati	PPMI	GE, SM, PL	1.5 T, 3 T	MPRAGE, FSPGR	N	HT, PD, ESP, HT	RS	31–83	100
(611 images in total)	ABIDE‐II	PL	3 T	MPRGAE, FSPGR	N	HT, AT	RS	5–64	100
	MIRIAD	GE	1.5 T	FSPGR	N	HT, PD	RS	55–80	64
	SRH	N/A	N/A	N/A	Y/N	EP	CL	N/A	42
External	IXI	GE, PL	1.5 T, 3 T	N/A	N	HT	RS	20–86	100
Test	LA5C	SM	3 T	MPRGAE	N	HT, BP, SZ, AHD	RS	21–50	100
(417 Images in total)	UNC	SM	3 T	MPRAGE, FLASH	N	HT	RS	22–68	100
	THP	SM, PL	3 T	MPRGAE	N	HT	RS	N/A	45
	CHL	GE, SM	3 T	FSPGR	Y/N	PD, ET, DT, PN	CL	44–70	20
	CUB	SM	N/A	MPRAGE	Y/N	PD	CL	37–73	39
	AUH	SM	3 T	MPRGAE	N	PD	CL	58 ± 6	13

*Note*: Three types of scanners are used for image acquisition, Siemens (SM), Philips (PL), and General Electric (GE). Among them, there are 1.5 and 3 tesla (T) scanners. A wide range of protocols were used for the image acquisition and can be categorized into three groups; MPRAGE, FSPGR, and FLASH. In some scans, gadolinium enhancement (Gado) was used. This is shown by yes (Y), if the gadolinium enhancement was performed otherwise No (N). The HCP datasets contains healthy (HT) subjects, OASIS3: HT, Alzheimer's disease (AD), and dementia (DT), ADNI: HT, AD, memory concern (MC), and cognitive impairment (CI), PPMI: HT, Parkinson's disease (PD), and prodromal (early stage Parkinson's disease ‐ ESP), ABIDE‐II: HT, and autism (AT), MIRIAD: HT, and AD, SRH: Epilepsy (EP), IXI: HT, LA5C: HT, bipolar disorder (BP), schizophrenia (SZ), and attention deficit hyperactivity disorder (AHD), UNC: HT, THP: HT, CHL: PD, essential tremor (ET), dystonia (DT), pain (PN), CUB: PD, AUH: PD. some of the datasets are downloaded from open source servers and are generated for a specific research project. We refer to them as research‐orientated datasets (research‐RS). Other datasets are collected from our clinical collaborators and we refer to them as clinical or CL. The age range of subjects used in this study are shown in the age column, and finally the number (counts) of data used from each dataset are shown in the last column. N/a means that the corresponding meta data was not available.

### 2.2 Preprocessing

All MR images in this study were resampled to 1 × 1 × 1 mm voxel spacing, 256 × 256 × 256 dimensions, and conformed to the same slice orientation, left‐posterior‐inferior (LPI) (Henschel et al., 2020). Additionally, all the default nnU‐Net preprocessing steps, such as data augmentation, and intensity normalization, were applied before any training. We kept all the nnU‐Net preprocessing to evaluate the additional effect of our preprocessing on network's performance in section 3.4.

### 2.3 Data annotation

We created a label file including the segmentation of the 30 selected brain structures in MNI space (ICBM 2009b Nonlinear Asymmetric) (Fonov et al., 2009). This label file is generated once, later, when we get the transformation matrix from the native space to the MNI space for each subject, we transform this label file to the native space of that subject.

The segmentation maps were combined from the CIT168, DISTAL and THOMAS atlases (Ewert et al., 2018; Su et al., 2019; Pauli et al., 2018). Each structure was initially resampled to the MNI space, and thresholded at 0.5 to generate a binary label. The lateral ventricles were manually segmented using the MNI template in the ITK‐SNAP toolbox^14^ (Yushkevich et al., 2006). The segmentation labels were combined into a single file, in which each structure has a corresponding index. The full name of the labels, and their origin are outlined in Table 2.

**TABLE 2:** The full name of the brain structures, their abbreviations, sources, and the label numbers are shown in this table. Brain mask is obtained using ANTs. For the brain structures, the following atlases are used: CIT, Distal, and Thomas

Full name	Abbreviation	Source	Label
Brain mask	BM	ANTs	1
Caudate nucleus–left	CA‐L	CIT	2
Caudate nucleus–right	CA‐R	CIT	3
Globus pallidus externus–left	GPE‐L	Distal	4
Globus pallidus externus–right	GPE‐R	Distal	5
Globus pallidus internus–left	GPI‐L	Distal	6
Globus pallidus internus–right	GPI‐R	Distal	7
Habenular nuclei–left	HN‐L	Thomas	8
Habenular nuclei–right	HN‐R	Thomas	9
Internal capsule–left	IC‐L	Distal	10
Internal capsule–right	IC‐R	Distal	11
Nucleus accumbens–left	NAC‐L	CIT	12
Nucleus accumbens–right	NAC‐R	CIT	13
Putamen–left	PU‐L	CIT	14
Putamen–right	PU‐R	CIT	15
Red nucleus–left	RN‐L	Distal	16
Red nucleus–right	RN‐R	Distal	17
Substantia Nigra, pars compacta–left	SNC‐L	CIT	18
Substantia Nigra, pars compacta–right	SNC‐R	CIT	19
Substantia Nigra, pars reticulata–left	SNR‐L	CIT	20
Substantia Nigra, pars reticulata–right	SNR‐R	CIT	21
Subthalamic nucleus–left	STN‐L	Distal	22
Subthalamic nucleus–right	STN‐R	Distal	23
Thalamus–left	THAL‐L	Thomas	24
Thalamus–right	THAL‐R	Thomas	25
Ventral lateral posterior nucleus of thalamus–right	VPL‐R	Thomas	26
Ventral lateral posterior nucleus of thalamus–left	VPL‐L	Thomas	27
Lateral ventricle–left	VENT‐L	Manual	28
Lateral ventricle–right	VENT‐R	Manual	29
Ventrointermediate nucleus of thalamus–left	VIM‐L	Thomas	30
Ventrointermediate nucleus of Thalamus–right	VIM‐R	Thomas	31

The subject T1w images were annotated with the generated label file using the atlas‐based method described in (Husch et al., 2018). In brief, we first applied N4‐bias field correction and skull stripping. Next, we used ANTs to calculate a non‐linear warp between the subject T1w image and the MNI reference image. Using this transformation, we warped the segmentation labels to the subject image, resulting in segmentation of the 30 brain structures in the subject's native image space. Additionally, we used ANTs to generate a whole brain mask. This brain mask was added to the 30 segmentation labels to improve the network performance by helping it locate the brain area. All the images were randomly selected from the original datasets. All the registrations were checked to ensure the results. If the registration pipeline failed on a subject and the generated segmentation labels were fully incorrect, then the subject's image was discarded, to avoid any incorrect training by the network.

### 2.4 LODO cross‐validation and testing

We used the nnU‐Net framework for the training (Isensee et al., 2021). The network is a U‐Net composed of an encoder‐decoder architecture with skip connections. We trained a 3D full resolution network with the batch size of 2 and patch size of [128128112]. The optimizer is Stochastic gradient descent with Nesterov momentum $\left(\mu =0.99\right).$ nnU‐Net performs a random 5‐fold cross‐validation during the training. For this study, we did not train the network with the default cross‐validation of nnU‐Net, instead, we performed a LODO cross‐validation. This was done to evaluate the generalizability of the network and cross‐dataset transportability. We trained seven different networks (seven folds), during the training. All subjects from one dataset were left out while subjects from the six other datasets were used for the training. The performance of the network was evaluated on the dataset that was left out. The procedure was repeated for all seven networks. Lastly, we ensembled all the folds to generate the final network. Any combination of the seven folds is possible. Selecting fewer folds will directly reduce computation time. In practice, two folds are sufficient to cover all the training data. We evaluated the performance of the final ensemble model (all folds) on seven unseen datasets(Test set).

### 2.5 Evaluation metrics

We evaluated the performance of the network using the Dice similarity coefficient (DSC) and the and average Hausdorff distance (AHD).

DSC is a widely used metric to evaluate the similarity between two segmentation tasks. Here, we compared the segmentations generated with our network with the registration‐based segmentation method (gold standard). We measured the DSC separately for each structure, considering the value of 1 for the structure and 0 for the rest of the image. Average DSC per subject is the average DSC of all its structures.

Equation 1 is the mathematical expression of the DSC, where ∣G∣ is the set of gold standard pixels and ∣P∣ is the set of network prediction pixels (Dice, 1945).

(1) $$\mathrm{DS}\left(G,P\right)=\frac{2|G\cap P|}{|G|+|P|}$$

Hausdorff distance is a metric to evaluate the longest distance between two sets of points. In the field of segmentation, it is used to compare the boundaries of two segmentation tasks (Equation 2).

(2) $$\mathrm{AHD}\left(G,P\right)=\frac{\left(D_{P\to G}+D_{G\to P}\right)}{2}$$

where,

(3) $$D_{P\to G}=\frac{1}{|P|}\sum_{p\in P}\min_{g\in G}∥p,g∥$$

∣G∣, and ∣P∣ are the sets of points in the gold standard and network prediction respectively. $D_{P\to G}$ is calculated in the same way as $D_{P\to G}$ (3). For this purpose, we measured the average Hausdorff distance (AHD) using SimpleITK Python Toolkit.^15^ Similar to the DSC, we measured the AHD for each structure separately.

### 2.6 Comparison to SynthSeg

DBSegment focuses on the segmentation of small deep brain structures that are rarely segmented by currently available deep learning‐based method. However, for the overlapping structures, we compared DBSegment to SynthSeg, a recent deep learning‐based method for brain structure segmentation (Billot et al., 2021). Therefore, we compared to the following SynthSeg evaluated structures: Caudate (CA), Putamen (PU), Lateral ventricles (VENT), Pallidum (PA) segmented by SynthSeg was compared to the union of Globus Pallidus Externus and Globus Pallidus Internus segmented by DBSegment, and the Thalamus (THAL) resulted from SynthSeg, was compared to the union of Thalamus, and its two sub‐parts: VIM and VPL, resulted from DBSegment. In total, we tested SynthSeg and DBSegment on 50 random images from the test set, including IXI, LA5C, UNC, THP, CHL, and CUB datasets.

### 2.7 Causal diagram

To address the question of why the DSC is lower in some data compared to the others, we decided to draw a causal diagram for our study (Figure 2).

This diagram is inspired by the scaffold causal diagram suggested by (Castro et al., 2020) for medical imaging workflows. We expanded the original diagram by adding factors affecting acquisition conditions such as scanner (manufacturer, field strength), acquisition protocol, and the use of gadolinium. In addition, we expanded the patient characteristics by including factors such as age and sex. Due to the limited meta data, we did not examine other factors such as ethnicity, handedness, and so forth. Our gold standard annotation (segmentation) was obtained by the registration‐based approach using ANTs. Any change in the image caused by other factors of the diagram, can affect the performance of ANTs, as well. However, to simplify the graph, we did not include any factor in annotation conditions for this study.

We used this diagram to extract the factors affecting the final annotation, as well as an ablation study to evaluate the effect of each factor on the final DSC. For each factor, we separated all the cross‐validation and test results into different classes. For instance, one of the factors affecting the annotation is the acquisition protocol. In this case, we separated the validation data into three protocol domains, including MPRAGE, FSPGR, and FLASH. This was to see if the performance on one domain differs compared to the others. We also measured the prevalence of each class in the training and test datasets to evaluate the existence of acquisition shift.

### 2.8 Preprocessing ablation study

In the ablation study, we evaluated the necessity of each preprocessing step. For this, we trained one network with no preprocessing, and four networks with different preprocessing steps, here referred to as preprocessing versions 1 to 4. In version 1, we perform only one preprocessing step, conforming all images to the same orientation, LPI. In version 2, we add one more step and conform all the images to the same orientation and voxel spacing (1 × 1 × 1 mm). In version 3, we conform all images to the same orientation, voxel spacing, and dimension (256 × 256 × 256). In version 4 we additionally normalize all images to the same intensity range (0–255). The last step is done only if the original maximum intensity value of the image is higher than 255. Regardless of the preprocessing version, all the default preprocessing of nnU‐Net were performed on the training data, including, intensity normalization (Z‐score per image), voxel spacing: resampling the images to the median of the training data, Dimension: if anisotropic: lowest resolution axis tenth percentile, other axes median, otherwise, median spacing for each axis, and data augmentation.

## 3 RESULTS

We present the results of the LODO cross‐validation per dataset, and per label with DSC and Hausdorff distance. The performance of the network was compared to the registration‐based method described in the methods section 2.3. Additionally, we evaluated the performance of the network on several datasets from different centers in the testing on unseen datasets section. Next, we performed an ablation study to evaluate the cross‐domain transportability of the network. Finally, we present another ablation study to evaluate the importance of the preprocessing steps.

### 3.1 LODO evaluation

**FIGURE 3:**
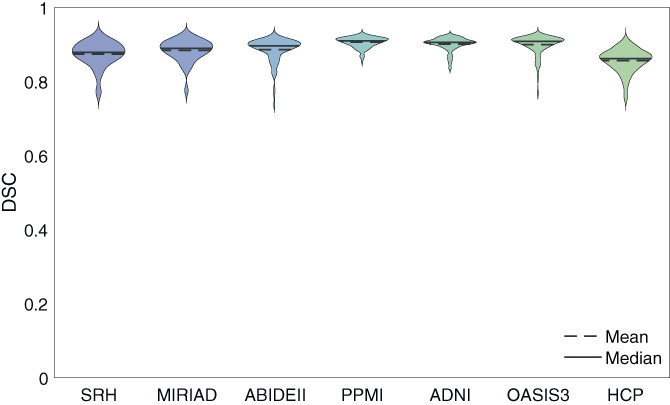
LODO cross‐validation performance. The performance of each fold was measured with DSC on the left out dataset. The average DSC of each subject was calculated for all labels excluding the brain mask and the ventricles. Subjects belonging to the same fold were plotted together. The average DSC of the folds were 0.87 ± 0.03, 0.88 ± 0.03, 0.89 ± 0.03, 0.91 ± 0.01, 0.90 ± 0.02, 0.90 ± 0.03, and 0.86 ± 0.03 on SRH, MIRIAD, ABIDE‐II, PPMI, ADNI, OASIS3, and HCP datasets respectively

The performance of each network on the left out dataset was compared to the gold standard (Figure 3). Each data point was calculated as the average DSC of a subject in the dataset. The number of subjects in each dataset are shown in Table 1. Networks showed similar performance on different datasets, with small variations seen as better performance on ADNI, PPMI, OASIS3, ABIDE‐II, and MIRIAD, and a slightly worse performance on the SRH, and HCP datasets. The average DSC of all cross‐validation subjects was 0.89±0.03, and the AHD across all cross‐validation subjects was 0.13±0.10.

**FIGURE 4:**
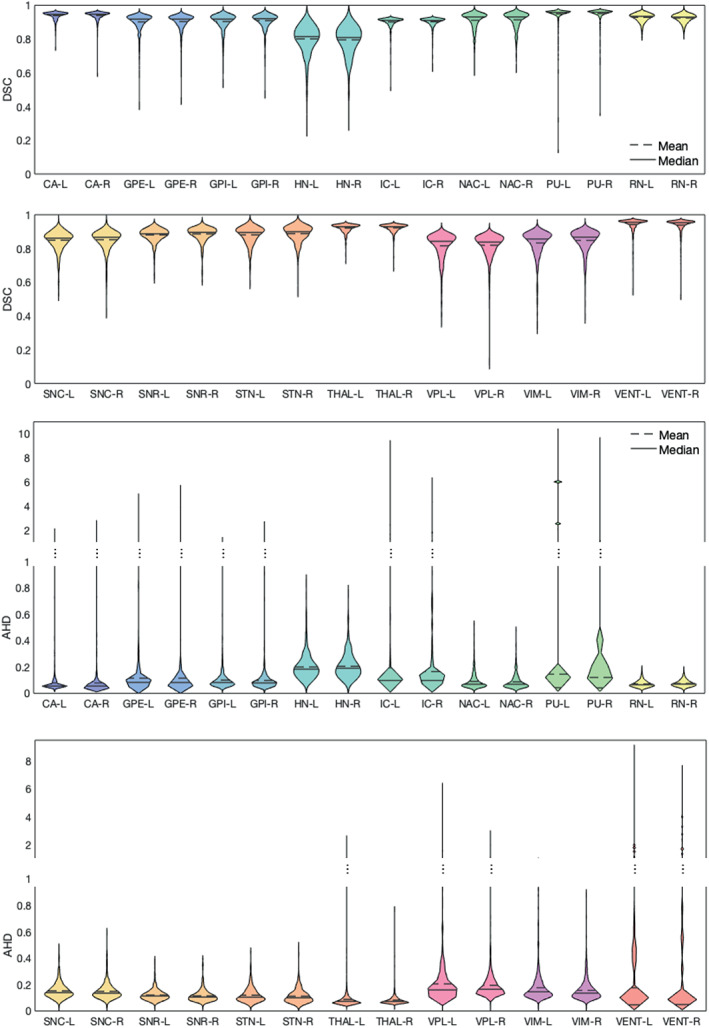
LODO cross‐validation performance on each label. On the top, the DSC of each label was measured for all the training subjects during LODO cross‐validation. Similarly, on the bottom, the AHD is measured. To visualize both the variability between different structures at lower AHD and the outliers at higher AHD values, the plot has two scales, below between 0 and 1 and on top between 1 and 9 and 1 and 10. The full name of the labels can be find in Table 2. The exact mean values of the DSC and the ADH are given in table S5

Figure 4 shows the DSC and the AHD per structure across all cross‐validation subjects. Better performance on large structures such as CA and THAL is observed compared to small structures, such as HN, VPL, and VIM.

### 3.2 Testing on unseen datasets

**FIGURE 5:**
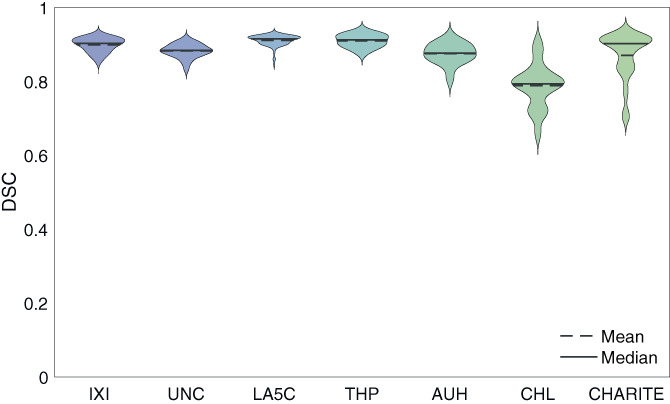
The network's performance on the unseen datasets (test sets). The average DSC of each subject was calculated for all its labels (all brain structures, without brain mask and the ventricles). The subjects belonging to the same dataset were plotted together. The average DSC of the datasets were 0.90 ± 0.02, 0.88 ± 0.02, 0.91 ± 0.01, 0.91 ± 0.02, 0.88 ± 0.03, 0.79 ± 0.06 and 0.87 ± 0.06 for IXI, UNC, LA5C, THP, AUH, CHL, and CUB respectively

The performance of the network was evaluated on 7 unseen datasets from different centers. The average DSC over all test subjects was 0.89±0.04, and the AHD was 0.12±0.07. Figure 5 shows the performance of the network on each dataset separately. Each data point in the violin plot is the average DSC of a subject in the dataset. There is less DSC between the network's output and the gold standard in the CHL and some of the CUB data, compared to the other datasets.

**FIGURE 6:**
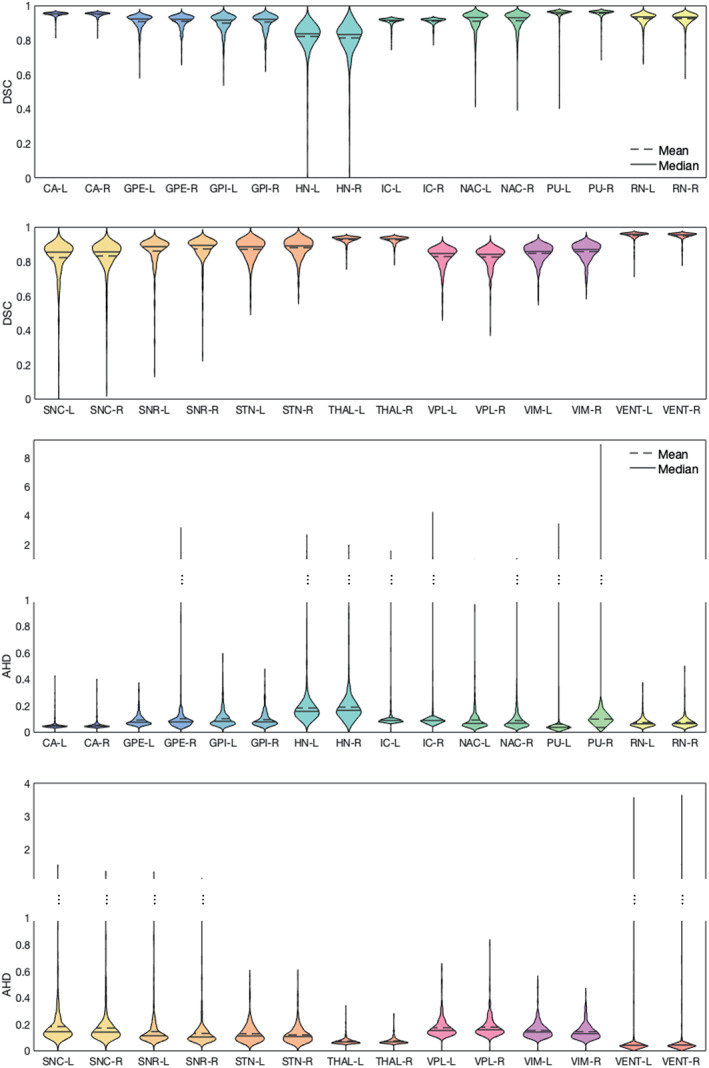
The network's performance on each label of the unseen datasets (test sets). On the top, the DSC of each label was measured across all the test subjects. Similarly, on the bottom, the AHD is measured. To visualize both the variability between different structures at lower AHD and the outliers at higher AHD values, the plot has two scales, below between 0 and 1 and on top between 1 and 5, and 1 and 9. The full name of the labels can be find in Table 2. The exact mean values of the DSC and the ADH are given in table S5

**FIGURE 7:**
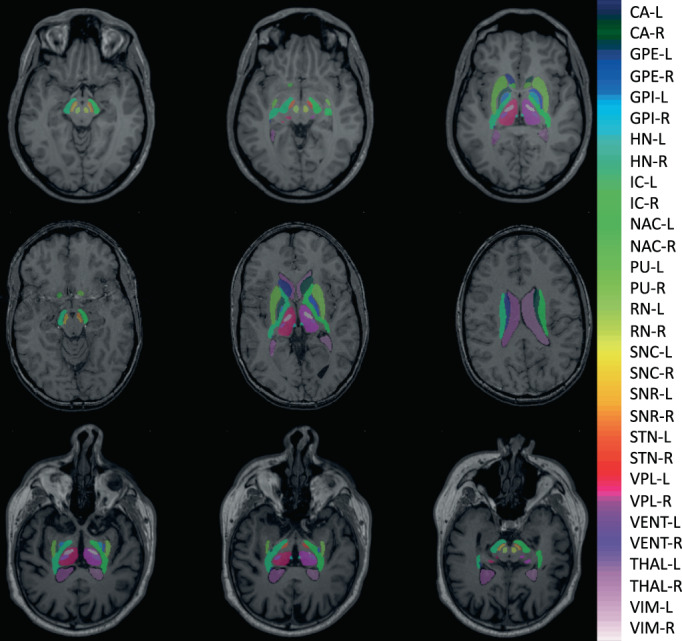
Examples of DBSegment outputs. Three random images from the test set are plotted with the segmentation out of DBSegment (rows). Three 2D slices are shown to for each subject (columns). Visible structures: Top right: IC, SNC, SNR, RN, top middle: STN, IC, RN, slightly visible: VENT, THAL, NAC, PU, top left: HN, THAL, VIM, VPL, IC, GPI, GPE, PU, NAC, VENT, middle right: IC, SNC, SNR, slightly visible: NAC, middle: THAL, VIM, VPL, HN, VENT, IC, PU, CA, GPE, slightly visible: GPI, middle left: VENT, CA, IC, bottom right: THAL, VIM, VPL, IC, VENT, PU, slightly visible: GPE, GPI, Bottom middle: THAL, HN, VPL, STN, IC, PU, slightly visible: VIM, GPE, bottom right: IC, SNC, SNR, VENT, RN, slightly visible: THAL, CA

In Figure 6, the DSC and AHD of all test data are shown per structure. Similar to the cross‐validation results, better performance is visible for bigger structures compared to the smaller structures. The number of outliers reduced compared to the cross‐validation. In Figure 7, examples of the network's segmentation outputs are visualized for three random data from the test set. In this figure, clear segmentation of small structures such as HN is visible.

### 3.3 Ablation study on cross‐domain transportability

**FIGURE 8:**
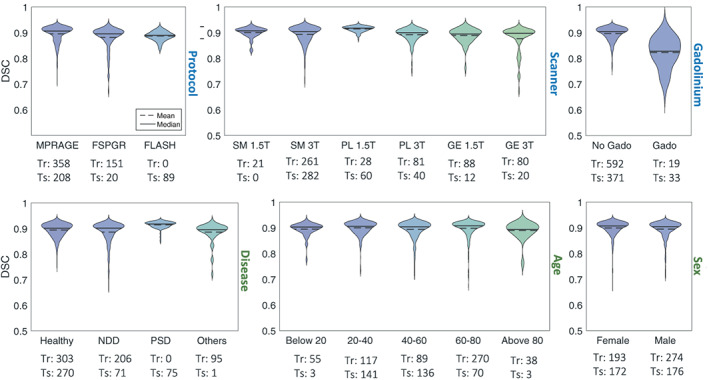
Analysis of DSC stratified by potential confounding factors. The factors were obtained from the causal diagram (Figure 2). Top row ‐ blue: Factors affecting the acquisition conditions. Protocol: MPRAGE with the mean DSC = 0.90 ± 0.03, FSPGR, mean DSC = 0.88 ± 0.04, and FLASH, mean DSC = 0.89 ± 0.02, scanner: Siemens (SM) 1.5 T, mean DSC = 0.90 ± 0.02, SM 3 T, mean DSC = 0.89 ± 0.03, Philips (PL) 1.5 T, mean DSC = 0.91 ± 0.01, PL 3 T, mean DSC = 0.89 ± 0.03, General Electric (GE) 1.5 T, mean DSC = 0.89 ± 0.03, and GE 3 T, mean DSC = 0.88 ± 0.05, gadolinium: Images without gadolinium enhancement (No Gado), mean DSC = 0.90 ± 0.03, and images with gadolinium enhancement (Gado), mean DSC = 0.82 ± 0.06. Bottom row ‐ green: Factors affecting the patient's characteristics. Disease: Healthy, mean DSC = 0.89 '± 0.03, neurodegenerative disorders (NDD), including, Alzheimer's disease, dementia, memory concern, cognitive impairment, Parkinson's disease, and early stage Parkinson's disease, essential tremor, and dystonia, mean DSC = 0.89 ± 0.04, psychiatric disorders (PSD), including bipolar disorder, schizophrenia, and attention deficit hyperactivity disorder, mean DSC = 0.91 ± 0.01, and others, including, epilepsy, autism, trauma, and pain, mean DSC = 0.88 ± 0.04. Age: Below and equal to 20, mean DSC = 0.89 ± 0.03, between 20 and 40 and equal to 40 (20–40), mean DSC = 0.89 ± 0.03, similarly 40–60, mean DSC = 0.89±0.03, 60–80, mean DSC = 0.90 ± 0.03, and above 80, mean DSC = 0.89 ± 0.03. Sex: Female, mean DSC = 0.90 ± 0.03, and male, mean DSC = 0.89 ± 0.03. The size of each class in the training set (Tr), and the test set (Ts) are shown on the bottom of the plots

We examine the influence of different factors (from the causal diagram) on the DSC between the network and the gold standard. All cross‐validation and test data were divided into different classes of each factor. For the acquisition conditions, we divided the data according to acquisition protocol (three classes: MPRAGE, FSPGR, FLASH), scanner (six classes: Siemens 1.5/3 T, Philips 1.5/3 T, GE 1.5/3 T), and the use of gadolinium (two classes: used or not) (Figure 8 ‐ blue background). For the subjects characteristics, we divided the data according to the disease (four classes: healthy, NeuroDegenerative Disorders‐NDD, and PSychiatric Disorders‐PSD, and others), age (five classes: below 20, 20–40, 40–60, 60–80, and above 80 years), and sex (two classes: female, and male) (Figure 8 ‐ green background). For all factors, including protocol, scanner, disease, age, and sex, the network demonstrated similar performance on different classes, indicating its generalizability across different domains, except in the gadolinium plot (Figure 8), which shows a clear reduction in the performance on images with high effects from the gadolinium enhancement.

At the bottom of the plot 8, the number of subjects from each class in the training data (Tr), and the test data (Ts) are shown. The number of subjects with the FLASH protocol during the training was zero, while there were 89 FLASH data in the test set. The network showed a good performance on the unseen protocol, indicating its domain adaptation. Similarly, no scan from subjects with psychiatric disorders was used during the training, while the network performed well on subjects with psychiatric disorders (e.g., LA5C dataset).

Furthermore, we evaluated the performance of the network on 53 scans from a dataset with 7 Tesla T1w data (ATAG‐7 T,^16^) (Keuken et al., 2013). The network output a reasonable segmentation (evaluated by an expert), while the registration‐based method failed. This shows the network's domain adaptation on the new ultra‐highfield imaging data, and the robustness of the method (Figure S12).

Another example of the method's robustness can be seen in cases where the scan consists of a half brain. In these cases, the network output reasonable segmentations (evaluated by an expert), while the registration‐based method failed (Figure S13).

### 3.4 Ablation study on the preprocessing

We evaluated the importance of different preprocessing steps used in this study. The average DSC across all cross‐validation data are compared between five networks, one with no preprocessing, and four networks with preprocessing version 1 to 4 (V1: LPI, V2: LPI, 1 mm voxel, V3: LPI, 1 mm voxel, 256 dimension, V4: LPI, 1 mm voxel, 256 dimension, 0–255 intensity; see method section for further information on the different versions 2.8).

**FIGURE 9:**
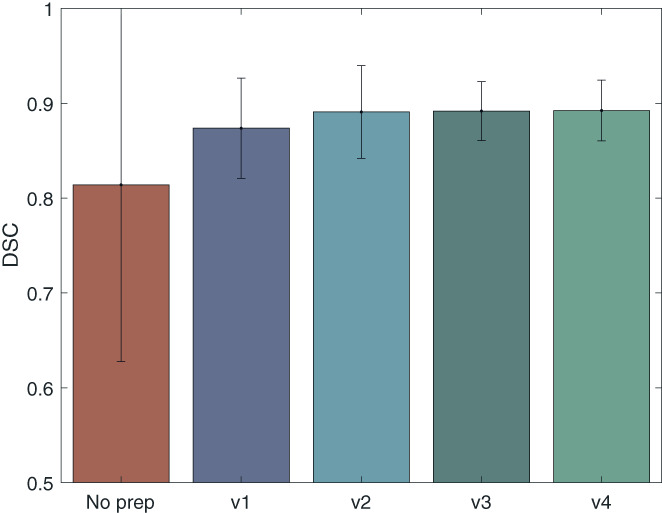
Results of the ablation study on the preprocessing steps. The DSC performance of five networks with different preprocessing steps are presented for all cross‐validation data. Standard deviations across subjects are presented on top of the bar plots. The first network is not using any preprocessing. The second network, builds on the V1 preprocessing, conforming all MR images to the same orientation. For the V2 preprocessing, all MR images were conformed to the same orientation and 1 × 1 × 1 mm voxel spacing, in V3, MR images were conformed to the same orientation, 1 × 1 × 1 mm voxel spacing and 256 × 256 × 256 dimension, in V4, MR images were conformed to the same orientation, 1 × 1 × 1 mm voxel spacing, 256 × 256 × 256 dimension, and the intensity range of each image was normalized between 0 and 255

There is a considerable difference between the network with no preprocessing and the network with the V1 preprocessing (Figure 9). The average DSC is 0.81 ± 0.19, 0.87 ± 0.05, 0.89 ± 0.05, 0.89 ± 0.031, 0.89 ± 0.032, for no preprocessing, V1, V2, V3, and V4, respectively. In Figure S11, we can see that the nnU‐Net alone fails to generalize on the MIRIAD dataset, which shows the importance of conforming all the MR images to the same orientation. The network with the V3 preprocessing shows the best performance compared to V1 and V2. It has a higher average DSC compared to V1 and lower variation compared to both V1 and V2. The difference between the V3 and V4 networks is negligible, meaning that normalizing the images between the intensity range of 0–255 is not necessary. We conclude that conforming the MR images to the same orientation, voxel spacing and the dimension (preprocessing V3), prior to the nnU‐Net default preprocessing, improves the performance of the network and the main improvement is obtained by the reorientation.

### 3.5 Brain mask

**FIGURE 10:**
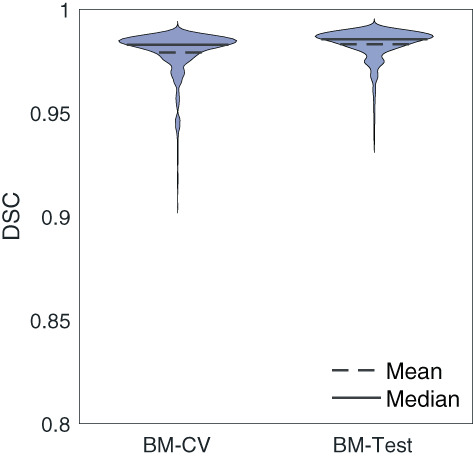
The DSC between the network and the gold standard brain mask. The DSC is plotted for all the cross‐validation subjects (BM‐CV), and all the test subjects (BM‐test)

In addition to the deep brain structures, the network outputs a brain mask. The brain mask was compared with the brain mask obtained by the ANTs brain extraction function of ANTs (Figure 10). The average DSC between the two brain masks was 0.98±0.01 among all the cross‐validation data and 0.98±0.01 among all the test data.

### 3.6 Time efficiency

The inference time of the network depends on the number of networks (folds) used for the ensemble. We provide seven different folds. Figure S14 shows the comparison between the performance of the network on 50 test images using different number of folds for the ensembling, as well as the time required for the inference respectively. We suggest an ensemble of two folds as a reasonable trade‐off between performance and low‐inference time.

Another factor affecting the inference time, is the computational power. Our method leverages the available GPU, while ANTs uses all available CPUs, therefore, to have a fair comparison, in Table 3, we compared the inference time of DBSegment and ANTs using different computational power. In the presence of a GPU, our method is ∼40 times faster than ANTs, and in the absence of a GPU, it is ∼13 times faster. For instance, with 8 CPU and 1 GPU performed on a node of a high performance computing cluster, the average time required for our network's inference was ∼1.3 min to output both the segmentation and the brain mask in the patient's space, while the registration‐based method (using ANTs) on average required ∼43 min for the segmentation. Additionally, the average time required for obtaining the brain mask with ANTs was ∼53 min. There are faster ways to obtain a less accurate brain masks (e.g., *bet* function of FSL), therefore, we did not consider the time ANTs required to obtain a brain mask in Table 3.

**TABLE 3:** Comparison of the time required for the inference of DBSegment and ANTs using different computational power. The values are reported in minutes

Computational power	DBSegment	ANTs
1 GPU, 2 CPU	2.6	149
1 GPU, 4 CPU	1.9	79
1 GPU, 6 CPU	1.4	56
1 GPU, 8 CPU	1.3	43
1 GPU, 16 CPU	1.1	30
0 GPU, 2 CPU	10.2	133
0 GPU, 4 CPU	5	74
0 GPU, 6 CPU	4.2	52
0 GPU, 8 CPU	2.6	42
0 GPU, 16 CPU	2.4	32

### 3.7 Comparison to SynthSeg

Table 4 shows the comparison of DBSegment and SynthSeg. overall, there is a high agreement between the segmentation map of SynthSeg and DBSegment on the overlapping structures. Most of the structures show a dice score similarity above 0.80, while PA shows the least DSC agreement of 0.70. Similarly, PA shows the largest AHD. On average, SynthSeg required 5 min to segment one image on a node of a high performance computing cluster with eight CPU and one GPU. With the same computational power, DBSegment required 1.3 min.

**TABLE 4:** DBSegment versus SynthSeg. The DSC and the AHD are shown with the corresponding standard deviations

Labels	DSC	AHD
CA‐R	0.83 ± 0.07	0.33 ± 0.15
CA‐L	0.83 ± 0.05	0.33 ± 0.08
PU‐R	0.87 ± 0.04	0.18 ± 0.05
PU‐L	0.86 ± 0.03	0.20 ± 0.05
PA‐R	0.70 ± 0.04	0.36 ± 0.09
PA‐L	0.70 ± 0.04	0.36 ± 0.09
THAL‐R	0.78 ± 0.03	0.26 ± 0.05
THAL‐L	0.80 ± 0.03	0.29 ± 0.05
VENT‐R	0.84 ± 0.04	0.30 ± 0.16
VENT‐L	0.86 ± 0.04	0.29 ± 0.19

## 4 DISCUSSION

We presented a deep learning‐based network segmenting 30 deep brain structures and a brain mask. We evaluated the performance of the network on diverse datasets in multiple steps, using a LODO cross‐validation, testing on unseen datasets, and different ablation studies. The network segments quickly with high accuracy and robustness.

### 4.1 Networks evaluation

In the results of the LODO cross‐validation (Figure 3), we observed a lower average DSC in the SRH and the HCP dataset. We suggest that the lower DSC in the SRH dataset is associated with the 19 subjects with the gadolinium scans. For the HCP dataset, we believe that the high quality of the data separates it from the other datasets. Our network, which is trained on six datasets with of a typical quality, is then evaluated on a high‐quality dataset, resulting in lower DSC. Given that the HCP dataset is used as a training data in the six other folds, we think that the final network can generalize to high‐quality data.

Examining the DSC plot for the structures, there was a better performance on large structures compared to small ones. This is expected, as DSC measures the volume similarly, and a slight difference in a small volume can lead to a considerable reduction in the DSC. We can observe more outliers in the LODO cross‐validation results in the AHD plots of the structures, compared to the test data. Most of the outliers, belong to cases where the one network solely confused the left and the right hemisphere. However, after ensembling more than one network, the results improved on the test set.

We evaluated the final ensemble network on seven additional unseen datasets. A lower average DSC and higher variability was observed in the CHL dataset and similarly in some of the CUB datasets, which we suggest is caused by the gadolinium enhancement. We plan to investigate the effect of gadolinium enhancement on such automated methods in our future studies.

When comparing our method to SynthSeg, we observed a lower DSC for PA compared to the other structures. This is expected, as our method segments GPI and GPE individually, with a border line for a clear separation. The border line has no associated label. While SynthSeg segmented PA as a whole structure, therefore, the separating border line is also labeled as the PA. This causes a considerable difference between the two segmentation maps, thus a lower DSC agreement.

### 4.2 High diversity in clinical data

We used a diverse dataset for training as well as testing. This dataset contained data from 10 publicly available research oriented datasets, together with data from 4 clinical centers. Higher variation was observed in the clinical data (AUH, CUB, CHL, and RSH) compared to the research oriented ones (Figures 3 and 5). Data from research‐oriented datasets appears to be more standardized than clinical data due to strict protocols and homogeneity of procedures. In clinical practice, however, patient, clinician and center variability is to be expected. For example, we qualitatively observed much more patient head tilt in clinical acquisitions. Therefore, the clinical data is expected to be more variable and harder to generalize an automated method from. In most previous studies, access to clinical data was limited. We note this as a strength of this study, because we used a clinical dataset for the training and three in the test set. This provides evidence that our method can be adapted for clinical practice.

Furthermore, our network resulted better on half brain images compared to the registration‐based method (Figure S13). This is expected as our deep‐learning based method analyses patch sizes of (128128112), while in a registration‐based method, the whole image is analyzed at once. Thus, our method is more robust towards incomplete scans.

### 4.3 Ablation study on cross‐domain transportability

As presented in our causal diagram (Figure 2), there are various factors affecting the final DSC. We separated the data to different classes of each factor to see if performance was particularly low in one class. However, to isolate effects of each factor, other factors should ideally remain constant. For instance, age and the disease are in a chain that makes them dependent to each other, so when we look at the age plot, and we observe slightly lower performance in the class “above 80,” this could be associated with the higher prevalence of diseases in older ages, especially neurodegenerative disorders, rather than age solely.

We also considered the existence of the confounders. For instance, the outliers in the GE 3 T scanner and the FSPGR protocol are probably caused by the effect of gadolinium in the CHL data (the CHL data are acquired with GE 3 T and FSPGR protocol, Table 1), rather than the type of scanner or the acquisition protocol.

### 4.4 Ablation study on the preprocessing

We observed a low DSC on the MIRIAD dataset compared to the other datasets, when we trained a network with no prior preprocessing and just the default preprocessings of the nnU‐Net (Figure S11). One of the main differences observed between the MIRIAD dataset compared to the other datasets was the orientation. Most of the data are orientated in this order: right/left, posterior/anterior, superior/inferior, while the data from the MIRIAD dataset have the last two axis swapped. Adapting the preprocessing strategy improved the performance on the MIRIAD dataset (Figure S11) (Henschel et al., 2020). This highlights the importance of the MR orientation in the method's generalization.

### 4.5 Limitations

We used the registration‐based method to annotate the training data. Getting manual segmentation for 1000 train and test data for 30 brain structures in 3D is very time consuming and expensive. Furthermore, the main goal of this study is to propose a substitute for the registration‐based method, therefore, comparison with the registration‐based method is fair and reasonable.

We use T1w MRI for training and did not consider additional sequences to this version of DBSegment for two reasons. Not all our datasets have a T2 sequence, and as we want to have a diverse dataset for training and testing, we decided to keep all the datasets, instead of using only the ones that have both T1 and T2. Furthermore, we did not want to have the constraint of having both T1 and T2 for the users, so that with having only a T1, the segmentation would be possible. However, we are planning to develop a multi‐spectral version of DBSegment, where the network can leverage information from different MRI sequences or even different imaging modalities.

## 5 CONCLUSION

In this paper we presented a method to segment 30 deep brain structures and a brain mask from T1w MRI scans. The method performed almost as well as the registration‐based method (DSC = 0.89±0.04), while reducing the required time significantly, and enhancing the robustness. Furthermore, we assessed the cross‐domain transportability by evaluating the performance of the network separately on different domains of the factors affecting the performance. Finally, we provide the method as an easy‐to‐use python package.

### 5.1 Code availability

The source code of the method is available under GPL license on https://github.com/luxneuroimage/DBSegment. An easy‐to‐use python package is available via *pip* (pip install DBSegment, see https://pypi.org/project/DBSegment/). In the toolbox, we also provided an additional network, where instead of segmenting Subthalamic Nucleus (STN) as a whole, the sub‐parts of STN, Sensorimotor, Limbic, and Associative are segmented. The trained model files are provided via https://webdav-r3lab.uni.lu/public/deep_brain_seg/deep_brain_seg_model_7f.zip.

## CONFLICT OF INTEREST

The authors declare no competing interests.

## Supporting information


**Figure S11** Ablation Study on the preprocessing separately on each LODO cross‐validation dataset. Network's performance after no preprocessing, preprocessing v1, v2, v3, and v4 are shown on the cross‐validation dataset of the seven‐trained models. For more information about the different versions of preprocessing, refer to section 2.8 For the plot on the top right, the network was trained on ABIDE‐II, ADNI, HCP, MIRIAD, PPMI, and OASIS3, while SRH dataset was used as the validation set. Similarly, other six trainings were performed while one of the datasets was left out as the validation. The average DSC are as follow: SRH: No Prep: 0.86 ± 0.03, V1: 0.86 ± 0.03, V2: 0.88 ± 0.04, V3: 0.88 ± 0.03, V4: 0.88 ± 0.03. MIRIAD: No Prep: 0.32 ± 0.13, V1: 0.85 ± 0.04, V2: 0.87 ± 0.11, V3: 0.89 ± 0.03, V4: 0.89 ± 0.04 . ABIDE‐II: No Prep: 0.86 ± 0.09, V1: 0.87 ± 0.09, V2: 0.89 ± 0.03, V3: 0.89 ± 0.03, V4: 0.89 ± 0.03 . PPMI: No Prep: 0.90 ± 0.02, V1: 0.90 ± 0.02, V2: 0.91 ± 0.01, V3: 0.91 ± 0.01, V4: 0.91 ± 0.01 . ADNI: No Prep: 0.88 ± 0.02, V1: 0.88 ± 0.02, V2: 0.90 ± 0.02, V3: 0.90 ± 0.02, V4: 0.91 ± 0.02 . OASIS3: No Prep: 0.90 ± 0.02, V1: 0.90 ± 0.03, V2: 0.91 ± 0.02, V3: 0.90 ± 0.03, V4: 0.90 ± 0.03. HPC: No Prep: 0.85 ± 0.04, V1: 0.84 ± 0.05, V2: 0.86 ± 0.03, V3: 0.86 ± 0.03, V4: 0.86 ± 0.03.
**Figure S12**: The result of the deep brain structure segmentation by the proposed network and the registration‐based method on a raw 7 T MRI scan from the ATAG‐7 T dataset. The proposed network resulted in a correct segmentation, while the registration‐based method failed to segment the 7 T MRI.
**Figure S13**: The result of the deep brain structures segmentation by the proposed network and the registration‐based method on an incomplete MRI scan from the CHL dataset. This data was not used in the test set as the gold standard label is incorrect. The proposed network resulted in a correct segmentation, while the registration‐based method failed to segment the incomplete scan.
**Figure S14**: Comparison of the inference time and the DSC of the performance when using different number of folds (networks) for the ensemble. The network performance was compared to the registration‐based method. For each number of folds, all the possible combinations were considered: 1 Fold: We used only one network (fold) to get the segmentation of 50 images. The test was run seven times, using fold 1 to fold 7 one at the time. The average time needed to get the segmentation of 1 image is shown in the plot. Fold 2: We used the ensemble of two networks (folds) to get the segmentation of 50 images. First with fold 1 and 2, then with fold 1 and 3, fold 1 and 4…, in total, we tested all 21 possible combinations of two folds out of 7 folds. The average time needed to get the segmentation of 1 image is shown in the plot. Fold 3: similarly, we segmented with all the possible combinations of ensembling 3 folds. In total 35 possibilities. Fold 4: 35 possibilities. Fold 5: 21 possibilities. Fold 6: 7 possibilities. Fold 7: Finally, we used one network, the ensemble of all 7 folds, to get the segmentation map.
**Table S5**: The labels' mean DSC and mean ADH are given with the standard deviation. CV refers to the results of the lODO cross‐validation and Test refers to the results of the test set.

## Data Availability

The data from the online datasets include the corresponding citation. The data from clinical centers are anonymized and are not publicly available.

## Footnotes

^1^

http://stnava.github.io/ANTs/

^2^

https://fsl.fmrib.ox.ac.uk/fsl/fslwiki/FLIRT

^3^

https://www.fil.ion.ucl.ac.uk/spm/software/spm12/

^4^

http://www.humanconnectomeproject.org/

^5^

http://fcon_1000.projects.nitrc.org/indi/abide/abide_II.html

^6^

www.ppmi-info.org

^7^

http://adni.loni.usc.edu/

^8^

https://www.oasis-brains.org/

^9^

http://miriad.drc.ion.ucl.ac.uk/

^10^

https://brain-development.org/ixi-dataset/

^11^

https://openfmri.org/dataset/ds000030/

^12^

https://www.insight-journal.org/midas/community/view/21

^13^

https://openneuro.org/datasets/ds000206/versions/1.0.0

^14^

www.itksnap.org

^15^

https://simpleitk.org/

^16^

https://www.nitrc.org/projects/atag_mri_scans/
