# Supplementary material for: DBSegment: Fast and robust segmentation of deep brain structures considering domain generalization
Source: Hum Brain Mapp. 2022 Oct 17;44(2):762–78. doi: 10.1002/hbm.26097 (PMC9842883; doi:10.1002/hbm.26097)
Supplement: Supplementary file 1 — Figure S11 Ablation Study on the preprocessing separately on each LODO cross‐validation dataset. Network's performance after no preprocessing, preprocessing v1, v2, v3, and v4 are shown on the cross‐validation dataset of the seven‐trained models. For more information about the different versions of preprocessing, refer to section 2.8 For the plot on the top right, the network was trained on ABIDE‐II, ADNI, HCP, MIRIAD, PPMI, and OASIS3, while SRH dataset was used as the validation set. Similarly, other six trainings were performed while one of the datasets was left out as the validation. The average DSC are as follow: SRH: No Prep: 0.86 ± 0.03, V1: 0.86 ± 0.03, V2: 0.88 ± 0.04, V3: 0.88 ± 0.03, V4: 0.88 ± 0.03. MIRIAD: No Prep: 0.32 ± 0.13, V1: 0.85 ± 0.04, V2: 0.87 ± 0.11, V3: 0.89 ± 0.03, V4: 0.89 ± 0.04 . ABIDE‐II: No Prep: 0.86 ± 0.09, V1: 0.87 ± 0.09, V2: 0.89 ± 0.03, V3: 0.89 ± 0.03, V4: 0.89 ± 0.03 . PPMI: No Prep: 0.90 ± 0.02, V1: 0.90 ± 0.02, V2: 0.91 ± 0.01, V3: 0.91 ± 0.01, V4: 0.91 ± 0.01 . ADNI: No Prep: 0.88 ± 0.02, V1: 0.88 ± 0.02, V2: 0.90 ± 0.02, V3: 0.90 ± 0.02, V4: 0.91 ± 0.02 . OASIS3: No Prep: 0.90 ± 0.02, V1: 0.90 ± 0.03, V2: 0.91 ± 0.02, V3: 0.90 ± 0.03, V4: 0.90 ± 0.03. HPC: No Prep: 0.85 ± 0.04, V1: 0.84 ± 0.05, V2: 0.86 ± 0.03, V3: 0.86 ± 0.03, V4: 0.86 ± 0.03. Figure S12: The result of the deep brain structure segmentation by the proposed network and the registration‐based method on a raw 7 T MRI scan from the ATAG‐7 T dataset. The proposed network resulted in a correct segmentation, while the registration‐based method failed to segment the 7 T MRI. Figure S13: The result of the deep brain structures segmentation by the proposed network and the registration‐based method on an incomplete MRI scan from the CHL dataset. This data was not used in the test set as the gold standard label is incorrect. The proposed network resulted in a correct segmentation, while the registration‐based method failed to segment the incomplete scan. Figure S14: Co [file HBM-44-762-s001.pdf]

Supplementary Material

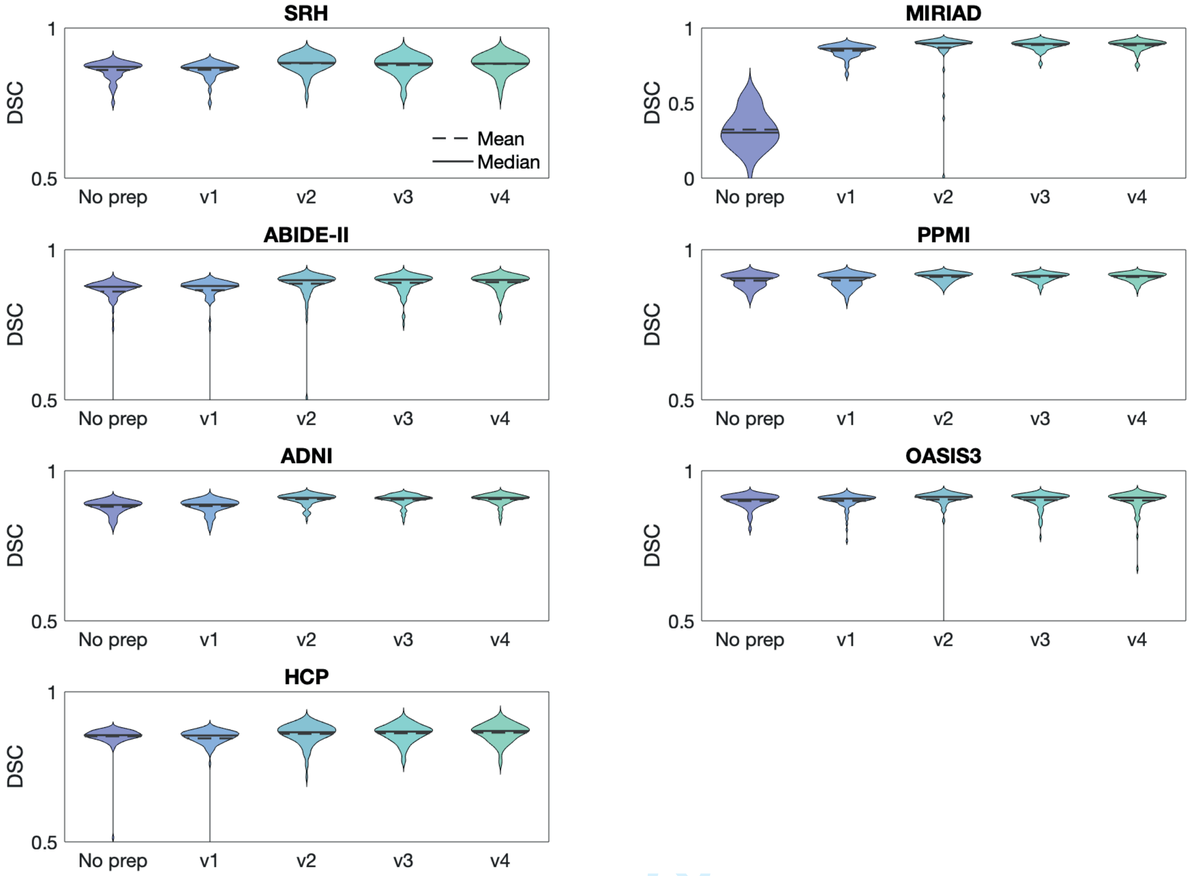

Figure S11: **Ablation Study on the pre-processing separately on each LODO cross-validation dataset.** Network's performance after no pre-processing, pre-processing v1, v2, v3, and v4 are shown on the cross-validation dataset of the 7 trained models. For more information about the different versions of pre-processing, refer to section 2.8 For the plot on the top right, the network was trained on ABIDE-II, ADNI, HCP, MIRIAD, PPMI, and OASIS3, while SRH dataset was used as the validation set. Similarly other 6 trainings were performed while one of the datasets was left out as the validation. The average DSC are as follow: SRH: No Prep: 0.86 ± 0.03, V1: 0.86 ± 0.03, V2: 0.88 ± 0.04, V3: 0.88 ± 0.03, V4: 0.88 ± 0.03 . MIRIAD: No Prep: 0.32 ± 0.13, V1: 0.85 ± 0.04, V2: 0.87 ± 0.11, V3: 0.89 ± 0.03, V4: 0.89 ± 0.04 . ABIDE-II: No Prep: 0.86 ± 0.09, V1: 0.87 ± 0.09, V2: 0.89 ± 0.03, V3: 0.89 ± 0.03, V4: 0.89 ± 0.03 . PPMI: No Prep: 0.90 ± 0.02, V1: 0.90 ± 0.02, V2: 0.91 ± 0.01, V3: 0.91 ± 0.01, V4: 0.91 ± 0.01 . ADNI: No Prep: 0.88 ± 0.02, V1: 0.88 ± 0.02, V2: 0.90 ± 0.02, V3: 0.90 ± 0.02, V4: 0.91 ± 0.02 . OASIS3: No Prep: 0.90 ± 0.02, V1: 0.90 ± 0.03, V2: 0.91 ± 0.02, V3: 0.90 ± 0.03, V4: 0.90 ± 0.03 . HPC: No Prep: 0.85 ± 0.04, V1: 0.84 ± 0.05, V2: 0.86 ± 0.03, V3: 0.86 ± 0.03, V4: 0.86 ± 0.03 .

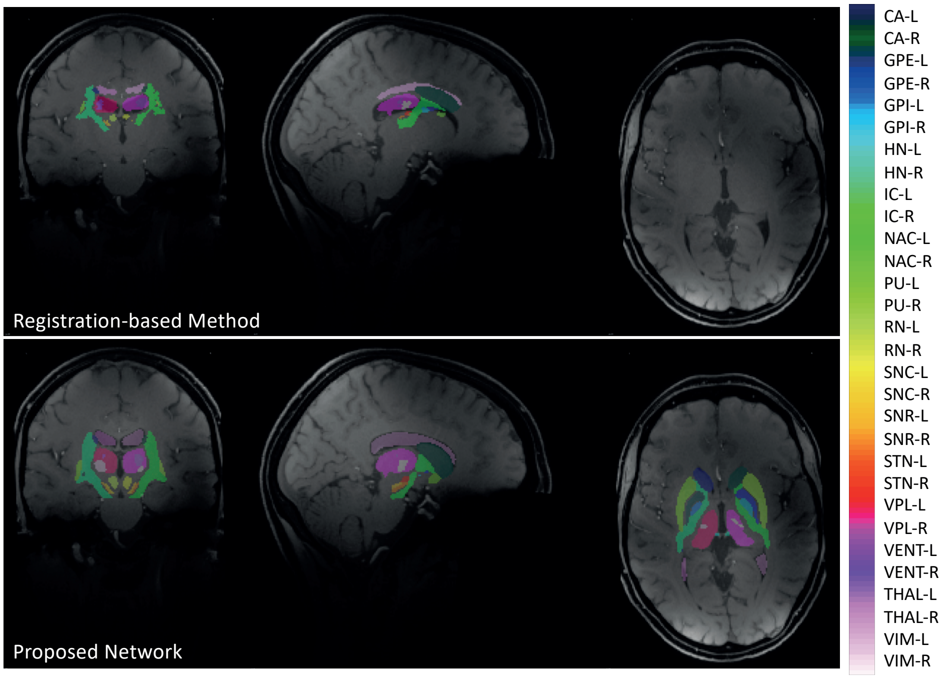

Figure S12: **The result of the deep brain structure segmentation by the proposed network and the registration-based method on a raw 7T MRI scan from the ATAG-7T dataset.**The proposed network resulted in a correct segmentation, while the registration-based method failed to segment the 7T MRI.

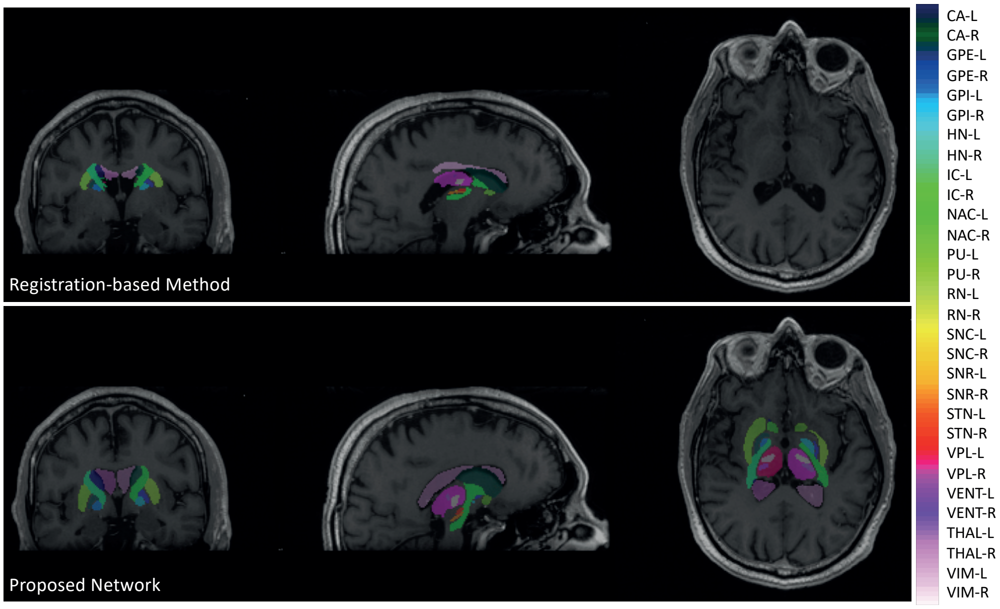

Figure S13: **The result of the deep brain structures segmentation by the proposed network and the registration-based method on an incomplete MRI scan from the CHL dataset**. This data was not used in the test set as the gold standard label is incorrect. The proposed network resulted in a correct segmentation, while the registration-based method failed to segment the incomplete scan.

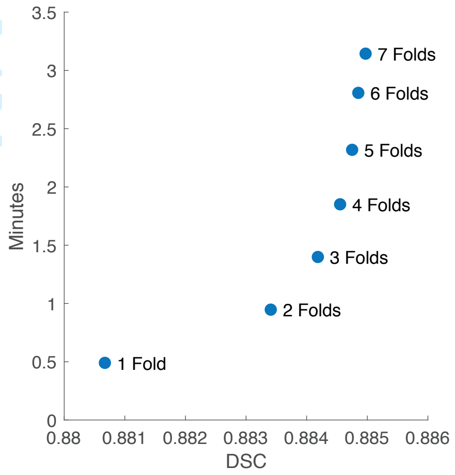

Figure S14: **Comparison of the inference time and the DSC of the performance when using different number of folds (networks) for the ensemble.** The network performance was compared to the registration-based method. For each number of folds, all the possible combinations were considered: **1 Fold:** We used only one network (fold) to get the segmentation of 50 images. The test was run seven times, using fold 1 to fold 7 one at the time. The average time needed to get the segmentation of 1 image is shown in the plot. **Fold 2:** We used the ensemble of two networks (folds) to get the segmentation of 50 images. First with fold 1 and 2, then with fold 1 and 3, fold 1 and 4..., in total, we tested all 21 possible combination of two folds out of 7 folds. The average time needed to get the segmentation of 1 image is shown in the plot. **Fold 3:** similarly, we segmented with all the possible combinations of ensembling 3 folds. In total 35 possibilities. **Fold 4:** 35 possibilities. **Fold 5:** 21 possibilities. **Fold 6:** 7 possibilities. **Fold 7:** Finally, we used one network, the ensemble of all 7 folds, to get the segmentation map.

Labels	DSC - CV	ADH - CV	DSC - Test	ADH - Test
CA-L	0.94 ± 0.02	0.08 ± 0.12	0.95 ± 0.02	0.05 ± 0.03
CA-R	0.94 ± 0.02	0.08 ± 0.16	0.95 ± 0.02	0.05 ± 0.03
GPE-L	0.90 ± 0.05	0.11 ± 0.22	0.91 ± 0.04	0.09 ± 0.05
GPE-R	0.90 ± 0.05	0.11 ± 0.28	0.91 ± 0.04	0.10 ± 0.17
GPI-L	0.90 ± 0.05	0.10 ± 0.08	0.90 ± 0.05	0.10 ± 0.06
GPI-R	0.91 ± 0.05	0.10 ± 0.12	0.91 ± 0.05	0.10 ± 0.05
HN-L	0.80 ± 0.09	0.20 ± 0.10	0.82 ± 0.10	0.18 ± 0.17
HN-R	0.80 ± 0.09	0.20 ± 0.09	0.81 ± 0.10	0.19 ± 0.15
IC-L	0.90 ± 0.03	0.19 ± 0.61	0.91 ± 0.02	0.11 ± 0.12
IC-R	0.90 ± 0.03	0.16 ± 0.43	0.91 ± 0.02	0.12 ± 0.28
NAC-L	0.91 ± 0.05	0.09 ± 0.06	0.91 ± 0.06	0.09 ± 0.08
NAC-R	0.91 ± 0.05	0.09 ± 0.06	0.91 ± 0.05	0.09 ± 0.07
PU-L	0.95 ± 0.05	0.14 ± 0.70	0.95 ± 0.04	0.06 ± 0.20
PU-R	0.94 ± 0.02	0.12 ± 0.68	0.96 ± 0.03	0.10 ± 0.66
RN-L	0.93 ± 0.03	0.07 ± 0.03	0.93 ± 0.04	0.07 ± 0.04
RN-R	0.95 ± 0.04	0.07 ± 0.03	0.93 ± 0.04	0.07 ± 0.04
SNC-L	0.85 ± 0.06	0.15 ± 0.06	0.82 ± 0.11	0.18 ± 0.14
SNC-R	0.85 ± 0.06	0.15 ± 0.06	0.83 ± 0.10	0.17 ± 0.13
SNR-L	0.88 ± 0.04	012 ± 0.04	0.86 ± 0.09	0.14 ± 0.12
SNR-R	0.89 ± 0.04	0.11 ± 0.05	0.87 ± 0.08	0.13 ± 0.10
STN-L	0.88 ± 0.05	0.12 ± 0.05	0.87 ± 0.06	0.13 ± 0.07
STN-R	0.89 ± 0.05	0.11 ± 0.05	0.88 ± 0.06	0.12 ± 0.06
THAL-L	0.92 ± 0.03	0.09 ± 0.11	0.93 ± 0.02	0.07 ± 0.03
THAL-R	0.92 ± 0.02	0.08 ± 0.05	0.93 ± 0.02	0.07 ± 0.03
VPL-L	0.82 ± 0.08	0.20 ± 0.28	0.83 ± 0.06	0.17 ± 0.07
VPL-R	0.82 ± 0.07	0.19 ± 0.16	0.83 ± 0.06	0.18 ± 0.07
VIM-L	0.83 ± 0.08	0.18 ± 0.11	0.85 ± 0.05	0.15 ± 0.06
VIM-R	0.85 ± 0.07	0.16 ± 0.08	0.86 ± 0.05	0.14 ± 0.06
VENT-L	0.94 ± 0.04	0.18 ± 0.56	0.95 ± 0.03	0.09 ± 0.28
VENT-R	0.94 ± 0.05	0.20 ± 0.63	0.95 ± 0.02	0.11 ± 0.36

Table S5: **The labels' mean DSC and mean ADH are given with the standard deviation. CV** refers to the results of the lODO cross-validation and **Test** refers to the results of the test set.

For Peer Review
